# Interaction Between *COMT* Genotype and MIND Diet, Cognitive Decline, and Incident Dementia: A Prospective Cohort Study

**DOI:** 10.3390/nu18193232

**Published:** 2026-09-30

**Authors:** Marilyn C. Cornelis, Rob M. van Dam, David A. Bennett, Julie A. Schneider

**Affiliations:** 1 Department of Preventive Medicine, Northwestern University Feinberg School of Medicine, Chicago, IL 60611, USA; 2 Department of Exercise and Nutrition Sciences, Milken Institute School of Public Health, The George Washington University, Washington, DC 20037, USA; 3 Rush Alzheimer’s Disease Center, Chicago, IL 60612, USA

**Keywords:** diet pattern, dementia, genotype, interaction

## Abstract

**Background:** Catechol-O-methyltransferase (COMT) metabolizes catecholamines and polyphenols unique to the Mediterranean-DASH Diet Intervention for Neurodegenerative Delay (MIND). The *COMT Val158Met* variant modified the effect of MIND on change in perceptual speed after 3 years in a randomized controlled trial. We aimed to evaluate and extend these findings to dementia incidence in a community-based prospective cohort. **Methods:** We included 745 older adults (mean age 80.0 years) from the Rush Memory and Aging Project. MIND adherence was estimated using a validated food-frequency questionnaire (0–15 points). Repeated cognitive assessments and dementia diagnoses were based on standardized clinical neurological examinations and criteria. Linear regression, linear mixed-effects, and Cox proportional hazards models examined associations of *COMT Val158Met* and its interactions with MIND with baseline cognition, cognitive decline, and incident dementia. Associations with neuropathology and COMT protein were also explored. **Results:** Significant *COMT* × MIND interactions were observed for Alzheimer’s (*p* = 0.003) and all-cause (*p* = 0.004) dementia and for *COMT* × MIND × time interactions with declines in global cognition (*p* = 0.008), episodic memory (*p* = 0.009), and visuospatial ability (*p* = 0.01). Cognitive benefits associated with MIND adherence were greater among *Met*/*Met* carriers (lower COMT activity). The hazard ratio (95% CI) per MIND point for Alzheimer’s dementia was 0.82 (0.69–0.98) for *Met*/*Met*, 1.00 (0.87–1.15) for *Met*/*Val*, and 1.05 (0.84–1.33) for *Val*/*Val*. Greater MIND adherence was associated with lower plasma COMT (*p* = 0.01), while each additional *Val* allele was associated with higher brain COMT (*p* < 0.0001). **Conclusions:** Individuals with genetically reduced COMT activity may be especially responsive to cognitive benefits of the MIND diet. Further study is needed to determine whether these effects differ across cognitive domains.

## 1. Introduction

Dementia develops over a prolonged preclinical period, creating an opportunity to identify modifiable factors that may influence cognitive trajectories before clinical disease becomes apparent. The Mediterranean-DASH Diet Intervention for Neurodegenerative Delay (MIND) was developed specifically for cognitive health and incorporates foods and nutrients drawn primarily from Mediterranean and DASH dietary patterns. Higher MIND adherence has been associated with slower cognitive decline and lower dementia incidence in several prospective studies, although findings have not been uniform [1,2,3,4,5]. Moreover, in a 3-year randomized trial, cognition improved among participants assigned to a calorie-restricted MIND intervention, but the improvement did not differ significantly from that observed with the calorie-restricted control diet [6].

Variation in biological response to diet could contribute to these heterogeneous findings. Genetic differences affecting the metabolism of dietary constituents provide one potential source of such variation. We recently investigated this possibility in a secondary analysis of the MIND randomized trial and identified an interaction between diet assignment and the functional *COMT* Val158Met variant for change in perceptual speed [7]. *COMT* encodes catechol-O-methyltransferase, which participates in the metabolism of catecholamines and several classes of polyphenols represented in MIND [8]. Participants homozygous for the *Met* allele, which is associated with lower COMT activity, showed greater improvement in perceptual speed with the MIND intervention than with the control diet, whereas this difference was not evident among *Val* allele carriers [7].

Whether this genotype-dependent association is evident for habitual MIND adherence and longer-term cognitive outcomes is unknown. We therefore examined *COMT* Val158Met and MIND adherence in the Rush Memory and Aging Project (MAP), a longitudinal community-based study with repeated cognitive evaluations, clinical dementia ascertainment, and postmortem neuropathologic assessment. We tested whether *COMT* genotype modified associations of MIND adherence with cognitive decline and incident Alzheimer’s and all-cause dementia. We additionally examined Alzheimer’s disease neuropathology and brain and plasma COMT protein abundance to explore potential biological correlates of the observed associations.

## 2. Methods

### 2.1. Rush Memory and Aging Project (MAP)

The Rush Memory and Aging Project (MAP) is an ongoing longitudinal cohort initiated in 1997 among older adults living in the Chicago metropolitan area [9]. Participants were dementia-free at enrollment and undergo annual clinical evaluations; all participants also consent to organ donation at death. Food-frequency questionnaires (FFQs) were incorporated into annual assessments between 2004 and 2013. During this period, 1306 existing or newly enrolled MAP participants were eligible for dietary assessment, which defined the baseline period for the present analysis. The study was approved by the Institutional Review Board of Rush University Medical Center.

### 2.2. Diet Assessment and MIND Adherence Score

Diet was assessed with a modified semiquantitative Harvard FFQ, previously validated among older adults in the Chicago area [10]. Approximately 15 food items contribute to the MIND adherence score [4] (Supplementary Table S1). Prespecified intake criteria assign 0, 0.5, or 1 point to each component, yielding a total score ranging from 0 to 15. The first available FFQ served as the primary dietary assessment.

### 2.3. Cognitive Assessments

At each annual visit, trained personnel administered a battery of 19 cognitive tests (Supplementary Table S2). Test scores were combined into measures of episodic memory, working memory, semantic memory, visuospatial ability, and perceptual speed, as well as an overall measure of global cognition. Individual test scores were standardized to the baseline MAP distribution before averaging them within each cognitive domain and across all tests for global cognition.

### 2.4. Dementia Outcomes

Dementia status was determined annually from the clinical evaluation. Alzheimer’s dementia was classified according to National Institute of Neurological and Communicative Disorders and Stroke and the Alzheimer’s Disease and Related Disorders Association [11,12], which require evidence of cognitive decline involving memory and at least one additional cognitive domain.

### 2.5. Genetic Data

The *COMT* Val158Met variant (rs4680) was assayed in subsets of MAP participants self-reporting as non-Hispanic white by whole-genome-sequencing (WGS) or genotyping on the Affymetrix 6.0 or Illumina OmniQuad Express platform as described previously [13]. For participants with data from both platforms, we prioritized WGS data over array-based data. *APOE* genotyping was performed independently using high-throughput sequencing.

### 2.6. Alzheimer’s Disease Neuropathology

Neuropathologic evaluations followed established MAP protocols [9]. Neuritic plaques, diffuse plaques, and neurofibrillary tangles were quantified across five cortical regions and combined into previously described summary measures, from which the global AD pathology measure was derived [14]. Additional outcomes included pathologic AD classified by National Institute on Aging (NIA)–Reagan criteria [15], modified Consortium to Establish a Registry for Alzheimer’s Disease (CERAD) classification based on neuritic plaque burden, and Braak staging (0-VI) reflecting the anatomical progression of neurofibrillary tangle pathology [16].

### 2.7. COMT Protein

COMT was extracted from proteomic profiles available for a subset of MAP participants. Dorsolateral prefrontal cortex (DLPFC) protein abundance was quantified using tandem mass tag mass spectrometry (TMT-MS) and expressed as relative protein abundance. Plasma protein abundance was quantified using the SomaScan 7K assay (SomaLogic, Inc., Boulder, CO, USA) and expressed as log2-transformed relative fluorescence units (RFU). Both methods have been described previously [17,18].

### 2.8. Other Covariates

Potential confounders were obtained from annual interviews and clinical assessments. These included education, income, smoking, late-life cognitive activity [19], depressive symptoms assessed with the 10-item Center for Epidemiologic Studies–Depression Scale (CESD) [20], physical activity based on the sum of self-reported minutes spent over the previous two weeks on specific activities [21]; body mass index (BMI), histories of hypertension, myocardial infarction, diabetes, and stroke [22]. Covariates were defined using previously established MAP procedures. Primary models used baseline values or values corresponding to the first FFQ; variables with repeated assessments were updated over time in analyses of cognitive decline.

### 2.9. Current Study Sample and Follow-Up Time

Of the 1064 MAP participants with a completed FFQ, 1054 of these had sufficient information to derive the MIND score and energy intake. We excluded 194 participants with no genetic data and 115 participants with less than 1 follow-up assessment or prevalent clinical dementia, leaving a maximal sample size of 745. WGS and array-based genetic data were available for 286 and 459 participants, respectively. The number of annual cognitive assessments analyzed for participants ranged from 2 to 23, with 77% of participants having five or more. Of these 745 participants, 651 died during follow-up, and 558 had neuropathological data. Analysis of brain and plasma COMT included 261 and 281 participants, respectively.

### 2.10. Statistical Analysis

All statistical analyses were performed using the SAS v9.4 statistical package (SAS Institute, Cary, NC, USA). We first examined the main effect of *COMT* genotype (with reference to *Met*/*Met*) on baseline cognition and cognitive decline using linear regression and linear-mixed models, respectively. Linear regression models included age, sex, and genotyping platform. Genotype was modeled as a continuous term to test for linear trends. Linear-mixed models with random intercept and time (slope) estimated mean differences in rates of cognitive decline over each time point by genotype. Models were constructed as described above, but each additionally included a variable for time and multiplicative terms between time and each model covariate; the latter providing the covariate-estimated effect on cognitive change. Cox proportional hazard regression models adjusted for age, sex, and genotyping platform were used to examine the association between *COMT* genotype and incident clinical all-cause and Alzheimer’s dementia. MAP participants were considered at risk for dementia from baseline and were followed up until the date of first clinical diagnosis or last clinical exam, whichever came first. The proportionality of hazards assumption was assessed using time-dependent explanatory variables and Schoenfeld residuals techniques [23] and was satisfied.

We next proceeded to test for *COMT* × MIND interactions for incident dementia and cognitive decline. To Cox models described above, we added MIND score and the cross-product term of *COMT* × MIND. Corresponding terms added to linear-mixed models include MIND × time, and *COMT* × MIND × time. A second model additionally controlled for education, late-life cognitive activity, *APOE* ε4 carrier status, income, smoking, alcohol intake, energy intake, depressive symptoms, physical activity, BMI, and histories of hypertension, diabetes, heart disease, and stroke; Cox models also included baseline global cognition. Repeatedly assessed cognitive and physical activity, BMI, depressive symptoms, and cardiovascular conditions were treated as time-varying covariates. Missing data, up to 5% for some covariates, were modeled as indicator variables. Given that *COMT* × MIND interactions were specified a priori based on findings from the MIND RCT [6], we defined statistical significance as *p* < 0.05. To account for multiple testing across the eight primary *COMT* × MIND interaction tests for cognitive decline and incident dementia, we additionally applied the Benjamini–Hochberg procedure to control the false discovery rate (FDR) at 5%. The primary analyses used MIND adherence derived from the first administered FFQ to preserve the prospective relationship between dietary assessment and subsequent cognitive outcomes. As a sensitivity analysis, we calculated the average MIND score across all available FFQs administered prior to incident dementia or the end of follow-up and repeated the *COMT* × MIND interaction analyses using this measure of longer-term MIND adherence. We note that the main effects of MIND adherence in MAP have been published previously [4,5] but we also present them for the current analytical sample for completeness. A MIND score excluding the wine component was derived to more closely align with the MIND RCT [6] but results were similar. Moreover, results were materially unchanged when models were additionally adjusted for the first five genetic ancestry principal components.

We explored the relationship between *COMT* and *COMT* × MIND with AD neuropathology (outcome) by performing logistic regression (NIA-Reagan or CERAD AD diagnosis), ordinal logistic regression (Braak stage), or linear regression (global AD pathology). We used a square root transformation of global AD pathology due to the positively skewed distribution of data. Similar covariate models described above were evaluated, but we replaced age at baseline with age at death (model 1) and added postmortem interval time (PMI, model 1) and clinical dementia status (model 2). Finally, multivariable regressions were used to explore the associations between (i) *COMT* genotype and brain/plasma COMT and (ii) the MIND score and brain/plasma COMT. Sample sizes were too small to explore associations between brain/plasma COMT and cognitive outcomes and neuropathology.

## 3. Results

### 3.1. Baseline Characteristics

Overall, the current MAP sample at baseline was 80.0 (SD = 6.7) years of age and 74% female. MAP participants with higher adherence to MIND were more likely to be younger, female, non-smokers, highly educated, free of a history of hypertension, diabetes or depression, and have lower BMI, and better cognitive ability (Table 1).

### 3.2. Main Effect of COMT Genotype on Baseline Cognition, Cognitive Decline and Incident Dementia

*COMT* was unrelated to baseline cognition after adjustment for age, sex, and genotyping platform. Relative to *COMT Met*/*Met*, *Met*/*Val* carriers showed slower (positive slope coefficients) rates of decline across several cognitive domains (Table 2), although a significant allele-dose trend was observed only for semantic memory (*p* = 0.02). During follow-up, 263 participants developed Alzheimer’s dementia and 273 developed all-cause dementia; neither outcome was associated with COMT genotype.

### 3.3. COMT × MIND Interactions for Cognitive Decline and Incident Dementia

In the current analytical sample, which is smaller compared to our previous reports [4,5], MIND adherence in the third tertile was associated with a lower rate of global cognitive decline [β = 0.03, 95% CI (0.009, 0.05), *p* = 0.004; *p* = 0.006 for trend] and a lower risk of Alzheimer’s [HR = 0.56, 95% CI (0.41, 0.76), *p* = 0.0002; *p* = 0.0002 for trend] and all-cause [HR = 0.55, 95% CI (0.40, 0.74), *p* = 0.0001; *p* = 0.0002 for trend] dementia compared to the first tertile after adjustment for age and sex. Full adjustment for *APOE* and confounders attenuated these results: MIND adherence in the third tertile was associated with a lower rate of global cognitive decline (β = 0.02, 95% CI (0.005, 0.04), *p* = 0.01; *p* = 0.01 for trend) and a non-significant decreased risk of Alzheimer’s [HR = 0.78, 95% CI (0.55, 1.10), *p* = 0.15; *p* = 0.29 for trend] and all-cause [HR = 0.78, 95% CI (0.56, 1.10), *p* = 0.16; *p* = 0.37 for trend] dementia compared to the first tertile.

Results for model 1 and model 2 tests of *COMT* × MIND interactions are reported in Supplementary Table S3. After adjusting for age, sex, genotype platform and other potential confounders (model 2), we observed significant *COMT* × MIND interactions for Alzheimer’s (*p* = 0.003) and all-cause (*p* = 0.004) dementia. In addition, we observed significant COMT × MIND × time interactions for declines in global cognition (*p* = 0.008), episodic memory (*p* = 0.009) and visuospatial ability (*p* = 0.01). All five interactions remained significant after correction for multiple testing using the Benjamini–Hochberg procedure (FDR-adjusted *p* = 0.016 for each). The interaction for perceptual speed was *p* = 0.05 (FDR-adjusted *p* = 0.067). *COMT*-stratified results are presented in Table 3. The associations between MIND adherence and better cognition were generally greater among those with the *Met*/*Met* genotype. Results were similar in sensitivity analyses using the average MIND score across repeated dietary assessments. COMT × MIND interactions remained significant for Alzheimer’s dementia (*p* = 0.002), all-cause dementia (*p* = 0.005), global cognition (*p* = 0.02), episodic memory (*p* = 0.04), and visuospatial ability (*p* = 0.04).

### 3.4. AD Neuropathology and COMT Protein Concentrations

The mean (SD) time between the first FFQ and death of MAP participants with neuropathology measures was 9.1 (4.7) years. *COMT* genotype was not directly associated with AD neuropathology, and no significant *COMT* × MIND interactions were observed. Post hoc analysis modeling MIND adherence assessed closest to death yielded similar results.

The mean (SD) time between the first FFQ and measures of plasma COMT was 2.2 (1.8) years. Measures of both brain and plasma COMT were available for only 83 participants and were not significantly correlated (Pearson r = 0.03, *p* = 0.76). Each *Val* allele associated with higher brain COMT levels after adjusting for age at death, sex, PMI, and genotyping platform (β = 2863 relative protein abundace, 95% CI (1576, 4149), *p* < 0.0001). *COMT* genotype was not associated with plasma COMT (*p* = 0.64). MIND diet adherence was inversely associated with plasma COMT (β = −0.008 log2-RFU, 95% CI (−0.014, −0.002), *p* = 0.01) but was not associated with brain COMT (*p* = 0.15). MIND adherence measured closest to death was also not associated with brain COMT (*p* = 0.20). Estimated mean brain/plasma COMT levels stratified by *COMT* genotype and MIND diet are presented in Supplementary Table S4. No single MIND component was driving associations with plasma COMT, and further adjustment for model 2 confounders yielded similar results. No significant *COMT* × MIND interactions for plasma or brain COMT levels were observed.

## 4. Discussion

Genetic differences are expected to explain some of the inter-individual variation in response to diet but defining specific gene–diet interactions has been challenging. The present findings provide independent support for a role of COMT genotype in modifying associations between the MIND diet and cognitive health. In MAP, COMT Val158Met modified associations of habitual MIND adherence with long-term decline in global cognition, episodic memory, and visuospatial ability and with risks of Alzheimer’s and all-cause dementia. Associations with greater MIND adherence were generally strongest among *Met*/*Met* carriers, consistent with the direction of the genotype-dependent response observed previously in the MIND RCT [7]. The current results therefore extend the earlier trial findings from short-term cognitive change to long-term cognitive trajectories and incident dementia in a community-based cohort.

Catechol-O-methyltransferase metabolizes catecholamines as well as specific polyphenols (i.e., hydroxytyrosol, anthocyanins) abundant in the MIND diet [8]. The most widely studied variant in *COMT* is the Val158Met substitution [24]. The *Met*/*Met* genotype creates a thermally less stable enzyme with lower activity; neurotransmitters such as dopamine and norepinephrine clear more slowly and maintain higher baseline availability in the prefrontal cortex [24,25,26,27,28]. The *Val* allele has been linked to a reduction of gray matter in dopamine-rich brain areas, which can worsen executive function and memory deficits [29]. The *COMT Val158Met* variant is the top SNP identified in GWAS of plasma (*N* = 34,557 to 67,761) [30,31] and brain (*N* = 268) [32] COMT concentrations. In a small subset of MAP participants with genetic data and measures of COMT, we confirmed the relationship of the genotype with brain COMT but not plasma COMT. In the MIND RCT, we reported the main effects of the *COMT* Val158Met variant with both baseline and 3-year change in executive function. *Val*/*Val* carriers had the lowest baseline score, but over 3 years, presented with the greatest score improvement. A link between this variant and executive function is consistent with prior studies [33,34]. Difference in change over time may be due, in part, to testing ceiling effects that mask any additional improvement among *Met* carriers. In MAP, *Val* was not associated with baseline cognition, but *Val* carriers presented with lower rates of decline in semantic memory and, to a lesser extent, global cognition, episodic memory, and visuospatial ability. Specific tests for executive function were administered to only a subset of MAP participants and at the time of first Alzheimer’s disease diagnosis. Working memory shares a common underlying executive attention component with executive function [35] but was not associated with *COMT* in MAP. Why the specific cognitive domains significantly impacted by *COMT* varied by study is unclear. Both studies administered cognitive tests in the clinic, but the battery of tests was not identical. Study enrollment criteria were also different, and both arms of the MIND RCT had a weight loss component. Whether our findings are specific to specific cognitive domains thus warrants further study.

Polyphenols can act as competitive substrates or mild inhibitors of the COMT enzyme [8,36,37,38]. In MAP, MIND adherence was inversely correlated with plasma COMT. This was also observed in a recent proteomic analysis of eight healthy diet patterns in the UK Biobank. COMT was significantly and inversely associated with all dietary patterns, but most strongly with MIND [39]. Methylation typically reduces the biological activity of a compound [8,40,41]. Thus, we hypothesized that *Met*/*Met* carriers may perform cognitively better on the MIND diet (rich in polyphenols) than *Val*/*Val* carriers since they benefit from genetically impaired COMT activity further compounded by the competitive or inhibitory actions of non-methylated (i.e., biologically active) polyphenols. In the MIND RCT, we observed a statistically significant COMT × diet interaction for 3-year change in perceptual speed. *Met*/*Met* carriers performed better on the MIND diet than the control diet. whereas no difference in change by diet was observed for *Val* carriers [7]. In the current analysis of MAP, we observed significant *COMT* ×MIND interactions for declines in global cognition, episodic memory, and visuospatial ability as well as for incident Alzheimer’s and all-cause dementia. Cognitive benefits of MIND adherence were greater among *Met*/*Met* carriers. Thus, in an RCT and community-based setting, our results suggest that *Met*/*Met* carriers respond better cognitively to the MIND diet than *Val* carriers. Again, cognitive domain specificity warrants further study. These observations are consistent with, but do not establish, a potential mechanism whereby genetically lower COMT activity among Met/Met carriers may alter the metabolism or biological availability of MIND-related polyphenols. The inverse association of MIND adherence with plasma COMT provides additional evidence of a relationship between diet and circulating COMT; however, MIND was not associated with brain COMT, and plasma and brain COMT were not correlated. The protein findings should be considered exploratory and do not establish that changes in COMT protein abundance mediate the observed *COMT* × MIND interactions.

We did not observe significant *COMT* × MIND interactions with measures of AD neuropathology. Several factors may contribute to this apparent difference from the clinical findings. Neuropathological analyses were restricted to the subset of participants who underwent autopsy and therefore had less statistical power to detect interactions. Moreover, the mean interval between the baseline dietary assessment and death was approximately 9 years, potentially increasing exposure misclassification, although analyses using MIND adherence measured closest to death yielded similar findings. It is also possible that COMT-related modification of the association between MIND adherence and cognition does not primarily operate through differences in classical AD neuropathology. COMT influences catecholamine and polyphenol metabolism, raising the possibility that the observed interaction relates to cognitive function through pathways other than plaque and tangle accumulation. However, the current analyses cannot distinguish among these mechanisms.

Strengths of the current study include the use of a well-characterized community-based aging cohort with detailed neurological assessments, standardized dementia ascertainment methods, post-mortem neuropathology, and extensive data on potential confounders. The design and participant characteristics of MAP differ from those of the MIND RCT (i.e., younger, overweight with a family history of dementia and suboptimal diet at trial enrollment), which supports the robustness of our overall findings. Nevertheless, we cannot discount residual confounding and measurement error for the dietary assessment. MAP participants were also White and not representative of the sampling population, and thus extrapolation of our findings to a more general and racially diverse population is limited.

## 5. Conclusions

Results of our genetic analysis of the MIND diet and cognitive health in the community-based MAP cohort align with those previously reported based on the MIND RCT [6]. Individuals with genetically impaired COMT activity may be especially responsive to the cognitive benefits of the MIND diet. If confirmed, these findings may have implications for personalized diets targeting cognitive health and for the design of future studies of the MIND diet.

## Figures and Tables

**Table 1:** Baseline characteristics of MAP participants by tertiles of MIND score ^1^.

Characteristic	T1	T2	T3
	3.5–7.0	7.5–8.5	9.0–13.0
	*N* = 274	*N* = 243	*N* = 228
Age, years	80.4 ± 7.1	80.8 ± 6.3	78.7 ± 6.5
Female, *n* (%)	201 (74)	179 (74)	174 (76)
Baseline cognitive function, Z-score	−0.05 ± 0.55	0.12 ± 0.50	0.29 ± 0.45
Mild cognitive impairment, *n* (%)	76 (28)	58 (24)	35 (15)
Education, years	14.2 ± 3.1	15.1 ± 2.7	15.6 ± 2.7
Hypertension, *n* (%)	207 (76)	176 (72)	155 (68)
Diabetes, *n* (%)	48 (18)	29 (12)	26 (11)
Stroke, *n* (%)	39 (14)	18 (7)	23 (10)
Heart disease, *n* (%)	56 (20)	33 (14)	39 (17)
Current smoker, *n* (%)	8 (3)	2 (1)	2 (1)
BMI, kg/m^2^	27.4 ± 5.0	27.3 ± 4.9	26.6 ± 5.3
^2^ Depressive symptoms, *n* (%)	17 (7)	8 (3)	3 (1)
MIND score	6.1 ± 0.8	8.0 ± 0.4	9.9 ± 0.9
APOE ε4 carriers, *n* (%)	57 (21)	47 (19)	49 (21)
^3^ *COMT* genotype	0.9 ± 0.7	1.0 ± 0.7	1.0 ± 0.7

^1^ Values are mean ± SD or *n* (%); ^2^ CESD-10 ≥ 5; ^3^ number of *Val* alleles.

**Table 2:** COMT genotype and Cognitive Decline ^1^.

COMT	Global Cognition		Episodic Memory		Visuospatial Ability		Perceptual Speed		Semantic Memory		Working Memory	
	β (95% CI)	*p*	β (95% CI)	*p*	β (95% CI)	*p*	β (95% CI)	*p*	β (95% CI)	*p*	β (95% CI)	*p*
*Met*/*Met*	Ref		Ref		Ref.		Ref.		Ref.		Ref.	
*Met*/*Val*	0.02 (0.0009, 0.04)	**0.04**	0.02 (0.002, 0.05)	**0.03**	0.02 (0.002, 0.03)	**0.02**	0.02 (0.0001, 0.03)	0.05	0.03 (0.007, 0.04)	**0.01**	0.01 (−0.004, 0.03)	0.13
*Val*/*Val*	0.02 (−0.002, 0.04)	0.08	0.02 (−0.008, 0.04)	0.18	0.01 (−0.006, 0.03)	0.19	0.01 (−0.008, 0.03)	0.24	0.02 (0.003, 0.05)	**0.03**	−0.003 (−0.02, 0.02)	0.77
Trend	0.01 (−0.0005, 0.02)	0.06	0.01 (−0.003, 0.02)	0.14	0.007 (−0.002, 0.02)	0.14	0.007 (−0.003, 0.02)	0.19	0.01 (0.002, 0.02)	**0.02**	−0.0007 (−0.01, 0.009)	0.89

^1^ Results from mixed linear models adjusted for age, sex and genotyping platform. Coefficients reflect change in cognitive function; a negative (positive) value corresponds to a decline (improvement) in cognitive function. Sample sizes for *Met*/*Met*, *Met*/*Val* and *Val*/*Val* genotypes are 198, 383 and 164, respectively. Bold-faced values are significant (*p* < 0.05).

**Table 3:** Associations between the MIND diet and cognitive health stratified by *COMT* genotype *.

	*Met*/*Met*		*Met*/*Val*		*Val*/*Val*	
	(*N* = 198)		(*N* = 383)		(*N* = 164)	
	HR (95% CI)	*p*	HR (95% CI)	*p*	HR (95% CI)	*p*
Alzheimer’s Dementia						
MIND T1	Ref.		Ref.		Ref.	
MIND T2	0.76 (0.41, 1.40)	0.38	0.85 (0.52, 1.38)	0.51	0.80 (0.35, 1.84)	0.60
MIND T3	0.33 (0.15, 0.74)	**0.007**	0.94 (0.53, 1.64)	0.81	0.71 (0.29, 1.75)	0.45
Trend (per point)	0.82 (0.69, 0.98)	**0.03**	1.00 (0.87, 1.15)	0.99	1.05 (0.84, 1.33)	0.66
All-cause Dementia						
MIND T1	Ref.		Ref.		Ref.	
MIND T2	0.79 (0.43, 1.46)	0.46	0.87 (0.55, 1.37)	0.54	0.72 (0.32, 1.63)	0.43
MIND T3	0.38 (0.17, 0.83)	**0.02**	1.00 (0.58, 1.73)	0.99	0.67 (0.28, 1.64)	0.38
Trend (per point)	0.84 (0.71, 1.00)	0.05	1.03 (0.90, 1.19)	0.65	1.03 (0.82, 1.28)	0.82
	β (95% CI)	*p*	β (95% CI)	*p*	β (95% CI)	*p*
Global cognition						
MIND T1	Ref.		Ref.		Ref.	
MIND T2	0.005 (−0.029, 0.040)	0.77	0.014 (−0.011, 0.040)	0.27	−0.026 (−0.059, 0.007)	0.13
MIND T3	0.033 (−0.002, 0.067)	0.07	0.019 (−0.007, 0.045)	0.15	0.003 (−0.032, 0.037)	0.87
Trend (per point)	0.010 (0.001, 0.017)	**0.01**	0.004 (−0.002, 0.011)	0.17	−0.002 (−0.011, 0.006)	0.59
Episodic memory						
MIND T1	Ref.		Ref.		Ref.	
MIND T2	0.007 (−0.037, 0.051)	0.76	0.012 (−0.020, 0.044)	0.47	−0.034 (−0.080, 0.013)	0.15
MIND T3	0.025 (−0.018, 0.069)	0.26	0.012 (−0.021, 0.044)	0.49	0.001 (−0.047, 0.050)	0.96
Trend (per point)	0.009 (−0.0003, 0.019)	0.06	0.003 (−0.005, 0.011)	0.45	−0.005 (−0.017, 0.006)	0.39
Visuospatial ability						
MIND T1	Ref.		Ref.		Ref.	
MIND T2	0.022 (−0.012, 0.056)	0.21	0.005 (−0.017, 0.027)	0.64	−0.009 (−0.038, 0.020)	0.54
MIND T3	0.018 (−0.015, 0.050)	0.29	0.007 (−0.015, 0.029)	0.53	0.007 (−0.022, 0.037)	0.61
Trend (per point)	0.005 (−0.003, 0.018)	0.21	0.003 (−0.002, 0.008)	0.26	0.0007 (−0.006, 0.008)	0.84

* Results from Cox (dementia outcomes) and mixed linear (cognitive change) models adjusted for age, sex, genotype platform, baseline global cognition (Cox models only), years of education, late-life cognitive activity, *APOE* carrier status, income level, cigarette smoking, alcohol consumption, energy intake, CESD score, physical activity, BMI and history of hypertension, diabetes, heart disease and stroke. Model 2 *p*-values for *COMT* × MIND interactions were 0.003 and 0.004 for Alzheimer’s and all-causes dementia, respectively. Model 2 *p*-values for *COMT* × MIND × time interactions were 0.008, 0.009 and 0.01 for global cognition, episodic memory and visuospatial ability, respectively. Bold-faced values are significant (*p* < 0.05).

## Data Availability

MAP data can be requested at https://www.radc.rush.edu.

## Supplementary Materials

The following supporting information can be downloaded at https://www.mdpi.com/article/10.3390/nu18193232/s1. Table S1: The Mediterranean-DASH Diet Intervention for Neurodegenerative Delay (MIND) Adherence Score Derivation. Table S2: Cognitive Tests Administered in MAP. Table S3: Results for significant tests of COMT × MIND interactions. Table S4: Plasma/brain COMT levels by *COMT* genotype and baseline MIND diet.
