# Supplementary material for: Interaction Between COMT Genotype and MIND Diet, Cognitive Decline, and Incident Dementia: A Prospective Cohort Study
Source: Nutrients. 2026 Sep 30;18(19):3232. doi: 10.3390/nu18193232 (PMC13666954; doi:10.3390/nu18193232)
Supplement: Supplementary file 1 [file nutrients-18-03232-s001.zip › nutrients-4572084-supplementary.pdf]

Supplementary Materials

Interaction Between COMT Genotype and MIND Diet, Cognitive Decline, and Incident Dementia: A Prospective Cohort Study

Marilyn C. Cornelis, Rob M. van Dam, David A. Bennett and Julie A. Schneider

Table S1. The Mediterranean-DASH Diet Intervention for Neurodegenerative Delay (MIND) Adherence Score Derivation.

Dietary component	0 points	0.5 point	1 point
Green leafy vegetables	≤2 servings/week	>2 to <7 servings/week	≥7 servings/week
Other vegetables	<5 servings/week	≥5 to <7 servings/week	≥7 servings/week
Berries	<1 serving/week	≥1 to <5 servings/week	≥5 servings/week
Nuts	<1 serving/week	≥1 to <5 servings/week	≥5 servings/week
Fish (not fried)	<1 serving/month or rarely	1-3 serving(s)/month or <1 serving/week	≥1 serving/week
Poultry (not fried, skinless)	<1 serving/week	≥1 to <2 servings/week	≥2 servings/week
Beans & legumes	<1 serving/week	≥1 to <3 servings/week	≥3 servings/week
Whole grains	<7 servings/week	≥7 to <21 servings/week	≥21 servings/week
Olive oil	<7 tablespoons/week	≥7 to <14 tablespoons/week	≥14 tablespoons/week
Wine	<1 glass/month or ≥2 glasses/day	1 glass/month to 1 glass/week	2-7 glasses/week
Butter and stick margarine	≥14 pats (teaspoons)/week	>7 to <14 pats/week	≤7 pats/week
Full-fat cheese	≥7 servings/week	>2 to <7 servings/week	≤2 servings/week
Red and processed meat	≥7 meals/week	≥4 to <7 meals/week	<4 meals/week
Fast and fried foods	≥4 meals/week	>1 to <4 meals/week	≤1 meal/week
Pastries & sweets	≥7 servings/week	≥5 to <7 servings/week	<5 servings/week

Table S2. Cognitive Tests Administered in MAP.

**Domain: Episodic Memory**
Word list memory
Word list recall
Word list recognition
East Boston story immediate
East Boston story delayed
Logical Memory Ia immediate
Logical Memory IIa delayed
**Domain: Semantic Memory**
Boston Naming Test
Verbal fluency
Reading test
**Domain: Working Memory**
Digit span forward
Digit span backward
Digit ordering
**Domain: Perceptual Speed**
Symbol Digit Modalities Test
Number comparison
Stroop word reading
Stroop color naming
**Domain: Visuospatial Ability**
Judgment of line orientation
Standard progressive matrices

Table S3. Results for significant tests of COMT × MIND interactions.

		Model 1*		Model 2†	
Outcome	Interaction tested	β (SE)	P	β (SE)	P
Alzheimer's dementia	*COMT*×MIND	0.14 (0.05)	0.007	0.17 (0.06)	0.003
All-cause dementia	*COMT*×MIND	0.12 (0.05)	0.02	0.15 (0.06)	0.004
Global cognition	*COMT*×MIND×Time	-0.007 (0.003)	0.03	-0.007 (0.003)	0.008
Episodic memory	*COMT*×MIND×Time	-0.009 (0.004)	0.02	-0.009 (0.004)	0.009
Visuospatial ability	*COMT*×MIND×Time	-0.006 (0.002)	0.009	-0.005 (0.002)	0.01
Perceptual speed	*COMT*×MIND×Time	-0.005 (0.003)	0.10	-0.005 (0.003)	0.05
Semantic memory	*COMT*×MIND×Time	-0.005 (0.003)	0.10	-0.005 (0.003)	0.08
Working memory	*COMT*×MIND×Time	-0.003 (0.003)	0.26	-0.003 (0.002)	0.22

*Adjusted for age, sex and genotype platform

†Adjusted for age, sex, genotype platform, baseline global cognition (dementia outcomes only), years of education, late-life cognitive activity, *APOE* carrier status, income, smoking, alcohol, energy intake, CESD score, physical activity, BMI and history of hypertension, diabetes, heart disease and stroke.

Table S4. Plasma/brain COMT levels by *COMT* genotype and baseline MIND diet.

Exposure	N plasma	Estimated mean (95% CI) plasma COMT, log2-RFU	N brain	Estimated mean (95% CI) brain COMT, relative protein abundance
COMT				
Met/Met	69	2.90 (2.88, 2.92)	61	12915 (11132, 14698)
Met/Val	145	2.90 (2.88, 2.91)	143	**16106 (14942, 17270)**
Val/Val	67	2.89 (2.87, 2.91)	57	**18737 (16890, 20585)**
MIND				
Tertile1	105	2.91 (2.89, 2.93)	109	15116 (13741, 16492)
Tertile2	94	2.90 (2.88, 2.92)	96	17005 (15535, 18475)
Tertile3	82	**2.88 (2.86, 2.90)**	56	15694 (13768, 17619)

Data are least squared means of plasma COMT adjusting for age, sex and genotyping platform or of brain COMT adjusting for age at death, sex, PMI and genotyping platform. Bold-faced values are significantly different from *Met/Met* (*COMT*) or Tertile1 (MIND)($p<0.05$). RFU: relative fluorescence units.
